# GPD1 inhibits the carcinogenesis of breast cancer through increasing PI3K/AKT-mediated lipid metabolism signaling pathway

**DOI:** 10.1016/j.heliyon.2023.e18128

**Published:** 2023-07-08

**Authors:** Zhengchao Xia, Ningming Zhao, Mingzhou Liu, DanDan Jiang, Shanjun Gao, Peizhi Ma, Li Huang

**Affiliations:** a Department of Pharmacy, Henan Provincial People's Hospital, Zhengzhou University People's Hospital, Zhengzhou, Henan, China; b Microbiome Laboratory, Henan Provincial People's Hospital, Zhengzhou, Henan, China; c Department of Pathology, Henan Provincial People's Hospital, Zhengzhou University People's Hospital, Zhengzhou, Henan, China

**Keywords:** Breast cancer, Glycerol 3-phosphate dehydrogenase 1, PI3K/AKT signaling pathway, Lipid metabolism, Biomarker

## Abstract

Glycerol 3-phosphate dehydrogenase 1 (GPD1) acts as a tumor suppressor in various types of cancer. However, the mechanisms of GPD1 anti-tumor remain unclear in breast cancer. This study aims to explore the function and clinical relevance of GPD1 in breast cancer. We confirmed that GPD1 inhibited the ability of proliferation, migration, and invasion in GPD1 overexpression breast cancer cells by CCK-8, wound healing, and Transwell assays, respectively. We found that GPD1 overexpression activated the lipid synthesis pathway and PI3K/AKT signaling pathway. The inhibitory effect of GPD1 on breast cancer cells was also weakened after treatment with LY294002, a PI3K/AKT pathway inhibitor. These results indicated that GPD1 suppressed the carcinogenesis of breast cancer through increasing PI3K/AKT-mediated lipid signaling pathways. Meanwhile, we detected that the relationship between GPD1 level and survival rate presents a positive correlation in breast cancer patients from the Cancer Genome Atlas (TCGA) database. Therefore, GPD1 can be a prognostic biomarker and target in developing therapeutic strategies for breast cancer patients.

## 1 Introduction

Breast cancer is the most common malignancy among women and the second cause of death through malignancies in women in developed countries [1]. Over the past decades, breast cancer patients have been offered a remarkable opportunity to elongate their lifespan along with an increasing number of diagnostic reagents and targeted medicine [[2], [3], [4]]. However, the prognosis and survival rate of patients have not dramatically improved [1,5]. The abnormal metabolism of breast cancer was mainly reflected in aerobic glycolysis, lipid metabolism, and various signaling pathways [[6], [7], [8]]. Among these metabolites, lipids have an advantage in tumor cell plasticity, therapeutic resistance, metastasis, and biomarkers [9]. Therefore, lipid regulatory pathways may be an effective therapeutic strategy and prognostic biomarker for breast cancer patients.

Glycerol 3-phosphate dehydrogenase 1 (GPD1) catalyzes the conversion of dihydroxyacetone phosphate derived from glucose to glycerol-3-phosphate (G3P) in the cytoplasm. G3P is the basic unit of various lipid metabolites, further serving as the backbone for lipid biosynthesis, the skeleton of cell membranes and different signaling molecules, participating in regulating biological processes of cell survival, energy metabolism, and oxidative stress [9,10]. Thus, GPD1 is considered a key element that connects carbohydrate and lipid metabolism [11,12]. It is reported that the mutation of the GPD1 gene caused many metabolic diseases in clinical cases, such as transient infantile hypertriglyceridemia, fatty liver, and obesity [13,14]. Accumulating evidence indicates that GPD1 also plays a suppressive role in various cancer, including lung cancer [15], renal clear cell carcinoma [11], glioblastoma [16], bladder cancer [17], and breast cancer [18,19].

Although relevant studies found that GPD1 has lower expression at both the transcriptional level and protein level in breast cancer cells, the functions of GPD1 remain largely unknown, particularly in lipid metabolism and signaling pathways. In this study, we explored the lipid regulatory pathway in breast cancer GPD1 overexpression cells and its underpinning mechanisms. We found that GPD1 overexpression up-regulated phosphatidylinositol 3-kinase (PI3K)/protein kinase B (AKT) signaling pathway and lipogenesis. Moreover, the composite biomarker models integrating GPD1 and lipid metabolites could robustly improve the prognostic accuracy or survival in breast cancer patients.

## 2 Material and methods

### 2.1 Cell culture and clinical specimens

The human breast cancer cell lines MCF-7, MDA-MB-231, and MCF 10A were purchased from the National Collection of Authenticated Cell Cultures (Shanghai, China). This study presents data on GPD1 expression and survival rates by distinguishing between patients with Luminal, HER2 positive, and triple-negative breast cancer (TNBC). The MCF-7 and MDA-MB-231 cell lines used were also derived from Luminal and triple-negative patients, respectively. All cells were cultured in Dulbecco's modified Eagle's medium (DMEM) (11965, Gibco, USA) supplemented with 10% fetal bovine serum (FBS) (10100, Gibco, USA), 100 U/ml penicillin G and 100 μg/mL streptomycin at 37 °C in a 5% CO_2_ humidified incubator [18].

The clinical samples of human breast cancer tissues and the corresponding adjacent benign tissues from the same patients were collected from Henan Provincial People's Hospital. The inclusion criterion was that the patient did not receive chemotherapy or radiotherapy before surgery. All patients were diagnosed and graded by pathologists to ensure the accuracy of clinical specimens. This study was approved by the Ethics Committee of Henan Provincial People's Hospital (Approval number: 2021-84). Informed consent was obtained from all subjects involved in the study.

### 2.2 Establishment of stable cell lines

Lentivirus packaging was provided by GenePharma Inc (Shanghai, China). A lentiviral vector plasmid LV5-EF1a-GFP + PURO was used in our study to construct the stable clones. The expression vector of GPD1 and negative control vector (pEX-3) were purchased from GenePharma Inc (Shanghai, China). Lipofectamine 2000 Transfection Reagent (11668, Invitrogen, USA) and Opti-MEM lower serum medium (31985070, Gibco, USA) were used to transfect the MDA-MB-231 cell lines according to the manufacturer's instructions. We further identified the effect of transfection in MCF-7 and MDA-MB-231 cell lines (MCF7-GPD1, 231-GPD1) by the RT-qPCR and western blotting assays.

### 2.3 Cell proliferation assay

Cell proliferation assays were performed by Cell Counting Kit-8 (CCK-8, CK04, Dojindo, Japan). Cells (1 × 10^4^ cells/well) were seeded into 6-well plates. 10 μL CCK-8 solution was added to each well at different time points respectively (4, 24, 48, and 72 h) after transfection. The plate was placed in the incubator for 2 h at 37 °C. The absorbance of the medium was detected by a microplate reader (Bio-Rad, USA) at 450 nm. All experiments were performed in triplicate.

### 2.4 Cell migration and invasion assays [11]

For cell migration assays, 5 × 10^5^ cells were seeded in the 6-well plate and placed in the incubator at 37 °C until approximately 90% confluence. After treated with medium without FBS for 24 h, a 200 μL sterile pipette tip was used to make scratches on each well. And the floating cells were removed with phosphate-buffered saline for 2 times. Photos were taken at 24 or 32 h to observe the healing of scratches using a microscope camera. The scratch healing area was calculated using Image J (Wayne Rasband, USA).

For cell invasion assay, 50 μL/well matrigel diluent (354234, BD Biosciences, USA) was coated into the upper compartment of the chamber, which was placed into the 24-well plate (3422, Corning, USA). After the matrigel solidification, the upper compartment of the chamber was seeded with 1 × 10^5^ cells suspended in 200 μL serum-free medium. The lower chamber was added with 500 μL DMEM containing 10% FBS. After incubation for 36 h at 37 °C, the upper chamber was washed with PBS, and the cells on the upper side of the membrane were removed with a cotton swab. The membrane was fixed with 4% paraformaldehyde for 20 min and dyed with 0.1% crystal violet at room temperature for 30 min. The photos of the invading cells were taken by the microscope camera. All assays were independently repeated triplicates.

### 2.5 RNA extraction and RT-qPCR

Total RNA was extracted using TRIzol (Thermo Fisher, USA) and chloroform reagent. After centrifugation, the RNA was precipitated with isometric isopropanol in the aqueous phase, and purified with 75% ethanol in DEPC water. The concentration and purity of RNA were determined using a NanoDrop 2000 spectrophotometer (Thermo Fisher, USA). The reverse transcription (RT) and the quantitative polymerase chain reaction (qPCR) were performed using the ReverTra RT Master Mix Kit (FSQ-301, Toyobo, Japan) and Thunderbird Next SYBR Green Master Mix (QPX-201, Toyobo, Japan) in the QuantStudio 5 Real-Time PCR System (Thermo Fisher, USA), following the manufacturer's protocol. The RNA expression levels were calculated using the 2^−ΔΔct^ method after normalization with the expression of GAPDH [10]. Sequences of all primers used for PCR were provided in Supplementary Table S1.

### 2.6 Western blotting

Total protein from MCF-7 and MDA-MB-231 cells was extracted by RIPA buffer (89900, Thermo Fisher, USA) supplemented with 1% protease inhibitor cocktail, 1% phosphatase inhibitor cocktail, and 1% PMSF (Sigma, USA). After centrifugation, the protein concentration was measured using a BCA Protein Assay Kit (P0012, Beyotime, China). Then the protein expression levels were examined by Western blot assay, which was separated by SDS-PAGE, transferred onto PVDF membranes, and incubated in different antibodies. The results were visualized with the PXi 9 chemiluminescent detection system (Syngene, England) and analyzed by using Image J. A list of antibodies is provided in Supplementary Table S2.

### 2.7 Lipidomics

MCF-7 cells and MCF7-GPD1 cells were harvested by trypsinization, rinsed twice in ice-cold PBS by low-speed centrifugation at 4 °C, and stored at −80 °C until analysis. The samples were extracted by the extraction solvent (methyl *tert*-butyl ether: methanol = 3:1, v/v) containing internal standard mixture on ice for 15 min and added into 200 μL water to centrifuge at 12,000 rpm for 10 min. 200 μL of the upper organic layer was collected and evaporated using a vacuum concentrator. The dry extract was reconstituted using 200 μL mobile phase B before LC-MS/MS analysis [10].

The full details of the lipidomics methods were explained in the Supplementary Material. In brief, the sample extracts were analyzed using ultra-performance liquid chromatography (UPLC)-ESI-MS/MS system (UPLC: ExionLC AD; MS: QTRAP, Sciex, USA). Differential metabolites (DMs) were identified in the Lipid Maps databases as the threshold of VIP (variable importance in projection) ≥ 1, *p*-value <0.05, and absolute log_2_FC (fold change) ≥ 1. Pathway enrichment analysis and function annotations of metabolites were performed using the Kyoto Encyclopedia of Genes and Genomes (KEGG) and HMDB database [10]. The experiment was completed by METWARE Inc (Wuhan, China).

### 2.8 Free fatty acid assay

The content of free fatty acid (FFA) in breast cancer cells was measured using commercially available kits (BC0595, Solarbio, China) according to the manufacturer's instructions. According to the protocol, cells (5 × 10^6^) were broken in 1 mL extracting solution by sonicator and centrifugated at 8000 rpm, 4 °C for 10 min. The upper phase was collected and placed in ice for the next step. A reaction system containing 30 μl samples, 300 μl reagent 1 (n-heptane: anhydrous methanol: chloroform = 24:1:25), and 120 μl reagent 2 (37 °C) oscillated for 10 min to centrifugate at 3000 rpm. A 50 μl upper liquid and 200 μl reagent 3 were mixed to oscillate for 2 min, and stand for 15 min. Finally, 200 μl samples were determined in 96-well plates to obtain the absorbance at 550 nm.

### 2.9 Bioinformatic analysis

The whole-transcriptome data and clinical characteristics of human breast cancer were acquired from the University of Alabama at Birmingham CANcer data analysis Portal (UALCAN, http://ualcan.path.uab.edu/). The mRNA expression levels and survival curves were drawn from the Cancer Genome Atlas (TCGA) database in the UALCAN based on breast cancer subclasses or individual cancer stages.

### 2.10 Statistical analysis

Statistical analysis was carried out using the SPSS version 22 (SPSS, Inc., Chicago, IL, USA) and GraphPad Prism 8. The experimental results were presented as the mean ± standard deviation (SD). The student's t-test and variance analysis (ANOVA) was used to analyze differences between two groups and multiple groups respectively. *P* < 0.05 indicated that the difference was statistically significant.

## 3 Results

### 3.1 GPD1 mRNA expression levels in breast cancer patients

To investigate the GPD1 mRNA expression levels in breast cancer patients, the box plots of GPD1 expression were drawn from the Cancer Genome Atlas (TCGA) database by the UALCAN. The RNA-seq data were grouped into primary tumor tissues (n = 1097) and normal tissues (n = 114) based on TCGA breast invasive carcinoma samples. We found that the GPD1 expression levels had an obvious decrease at 1.51 (median) in the tumor group compared with the normal group at 192.53 (median, *p* < 0.001, Fig. 1A). Meanwhile, the GPD1 expression levels declined to 1.68, 0.90, and 0.62 in the Luminal, HER2 positive, and TNBC subclasses, respectively (*p* < 0.001, Fig. 1B). Based on individual cancer stages, the GPD1 expression levels were also reduced to 3.74, 1.21, 1.67, and 0.85 in breast cancer compared with that in the normal group (*p* < 0.001, Fig. 1C). From these data, we discovered that the median level of GPD1 mRNA expression was significantly upper in the Luminal subtype vs. HER2 positive and TNBC subclasses separately (*p* < 0.001, Fig. 1B). Analogously, the GPD1 expression level was lower in stage 4 grade vs. stage 1 and stage 3, respectively.

**Fig. 1:**
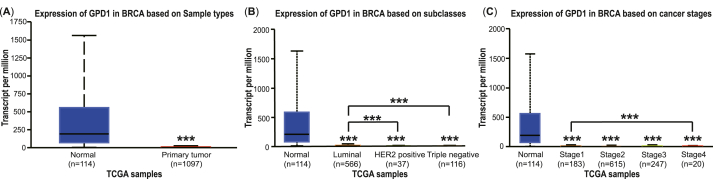
The GPD1 mRNA expression levels of breast cancer patients in TCGA. (A) GPD1 expression levels in breast invasive carcinoma and normal tissues. (**B**) GPD1 expression levels in different subtypes of breast cancer, including Luminal, HER2 positive, and TNBC. (**C**) GPD1 expression levels in four pathological stages of breast cancer.

### 3.2 The proliferation, migration, and invasion were inhibited in overexpressed GPD1 breast cancer cells

To verify the GPD1 mRNA levels in clinical tissues, 50 paired samples of breast cancer (tumor tissues and the adjacent normal tissues) were processed by RT-qPCR. The result showed that the GPD1 expression level was significantly downregulated in the tumor tissues compared with the normal tissues (*p* < 0.001, Fig. 2A). The mRNA and protein expression levels of GPD1 in breast cancer cells were examined by RT-qPCR and western blotting. As shown in Fig. 2B–C, GPD1 expression levels visibly declined in MCF-7 and MDA-MB-231 cell lines compared with the normal breast cells (MCF-10A, Fig. S9).

**Fig. 2:**
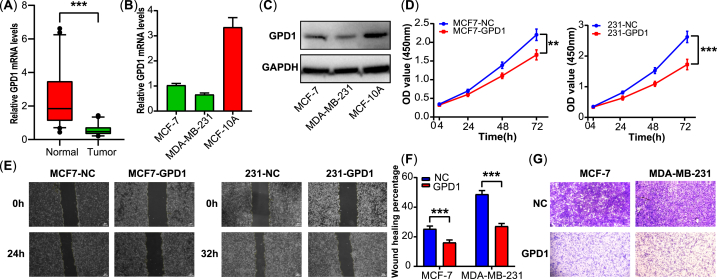
GPD1 overexpression inhibited cell proliferation, migration, and invasion. (A) The mRNA expression levels of GPD1 in breast cancer tissues (n = 50). (**B–C**) GPD1 expression in breast cancer cells and normal breast cells. (**D**) Cell proliferation as determined by CCK-8. (**E-F**) Cell migration as determined by wound healing assay. (**G**) Cell invasion as determined by Transwell assay. The data are presented as the mean ± SD. A two-tailed Student's t-test was used. *p < 0.05, **p < 0.01, ***p < 0.001.

According to the lower expression level of GPD1, we established the GPD1 overexpression breast cancer cell lines (MCF7-GPD1 and 231-GPD1) via lentiviral vector and plasmid vector transduction, respectively. The CCK-8 assay revealed that the overexpression of GPD1 inhibited cell proliferation in MCF-7 and MDA-MB-231 breast cancer cells (Fig. 2D). We further determined the effect of GPD1 on the ability of migration and invasion in breast cancer cells. The wound healing assays indicated that the overexpression of GPD1 suppressed the migration capability in MCF-7 and MDA-MB-231 cells (Fig. 2E–F). Consistent results showed that the capability of invasion was also weakened in the GPD1 overexpression group compared with the normal control group by Transwell assays (Fig. 2G).

### 3.3 Lipid metabolism was promoted with the overexpression of GPD1

A total of 1310 lipids were detected using LC-ESI-MS/MS, including glycerophospholipids (919), glycerides (189), sphingolipids (122), fatty acyls (55), sterol lipids (24) and prenol lipids (1), detailed data shown in Table S3 and Fig. S1. Compared with the normal control group, 80 up-regulated DMs and 30 down-regulated DMs were filtered with VIP and FC in the GPD1 overexpression group, involving 58 up-regulated and 21 down-regulated DMs in glycerophospholipids, 16 up-regulated and 2 down-regulated DMs in glycerides, 1 up-regulated and 6 down-regulated DMs in sphingolipids, 4 up-regulated and 1 down-regulated DMs in sterol lipids (Fig. 3A, S2 and Table S4).

The result of KEGG annotations and pathway enrichment analysis of DMs were shown in Table S5 and Fig. S3. The bubble chart of KEGG pathway enrichment contained the choline metabolism, Arachidonic acid metabolism, Linolenic acid metabolism, cholesterol metabolism, etc (Fig. 3B). In these signaling pathways, the phosphatidylinositol signaling pathway, phospholipase D signaling pathway, cAMP signaling pathway, and sphingolipid signaling pathway had up-regulated or down-regulated in Figs. S3 and S4.

**Fig. 3:**
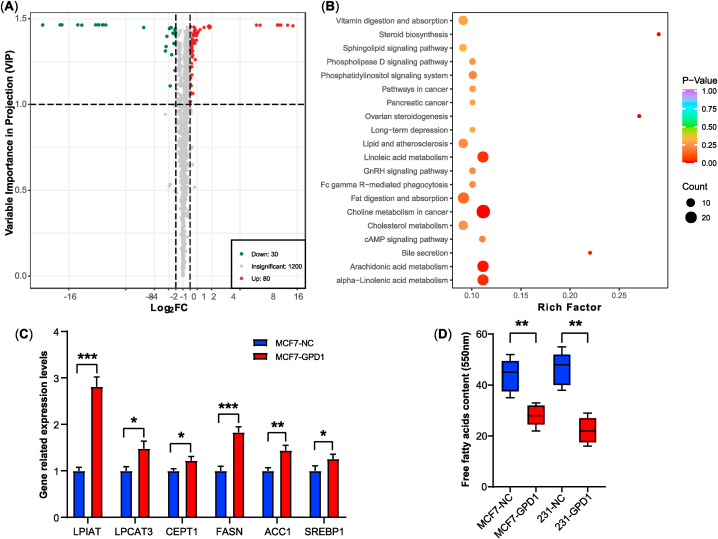
Overexpression of GPD1 up-regulated lipid metabolism. (A) The volcano map of different metabolism. Green and red dots indicated down- and up-regulated DMs, respectively. The abscissa represented the logarithmic value of the fold change (log_2_FC). The ordinate represented the VIP value. (**B**) The KEGG enrichment pathway of DMs. The abscissa represented the rich factor corresponding to each pathway. The color and size of the bubble points indicated the p-value and the number of enriched DMs, respectively. (**C**) The mRNA expression levels of the key lipid metabolic genes. (**D**) The contents of free fatty acids. Data are shown as the mean ± SD (n = 5, *p < 0.05, **p < 0.01, ***p < 0.001). (For interpretation of the references to color in this figure legend, the reader is referred to the Web version of this article.)

As the increased lipid metabolites content in the GPD1 overexpression group, we examined several key genes in lipid metabolism and signaling pathways including lysophosphatidylinositol acyltransferase (LPIAT), lysophosphatidylcholine acyltransferase (LPCAT3), choline/ethanolamine phosphotransferase (CEPT1), fatty acid synthase (FASN), acetyl-CoA carboxylase (ACC1) and sterol regulatory element-binding protein (SREBP1). The mRNA expression levels of related genes were remarkably increased compared with the normal control group (Fig. 3C). However, the partly descended free fatty acids levels in the GPD1 overexpression group indicated that more free fatty acids were used to synthesize lipid metabolites (Fig. 3D).

### 3.4 PI3K/AKT signaling pathway was increased in GPD1 overexpression cells

Related studies have reported that the PI3K/AKT signaling pathway plays an important role in the regulation of tumor development and metabolism, including lipid metabolism [20,21]. Therefore, we tested whether GPD1 affects PI3K/AKT signaling in MCF-7 and MD-MBA-231 cells. The Western blot analysis was used to evaluate the expression levels of PI3K, AKT, GSK3β, PTEN, and the phosphorylation levels of AKT at Ser473 and GSK3β at Ser9 in breast cancer cells (Fig. S10). As shown in Fig. 4A and B, we found that GPD1 overexpression enhanced the expression levels of PI3K, *p*-AKT, and *p*-GSK3β.

**Fig. 4:**
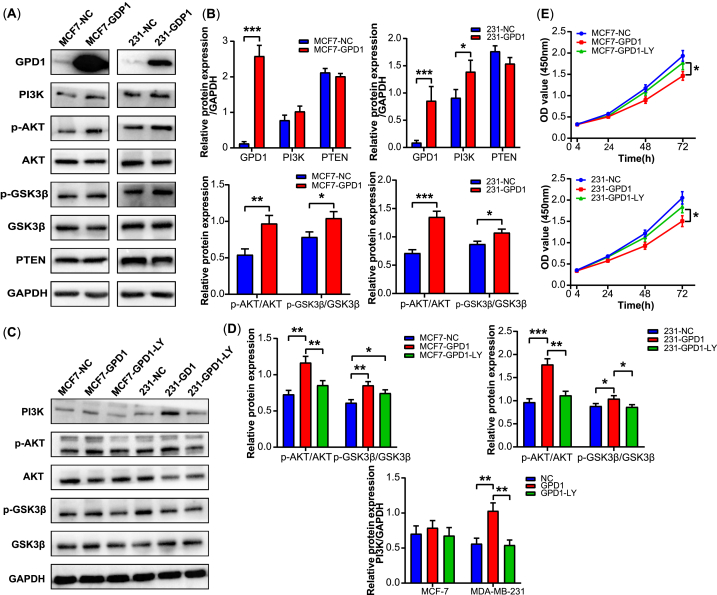
GPD1 overexpression activated PI3K/AKT signaling pathway. (A–B) The protein expression levels of PI3K, *p*-AKT, AKT, *p*-GSK3β, GSK3β, PTEN, and GAPDH after GPD1 overexpression. (**C-D**) The protein expression levels of PI3K, AKT, *p*-AKT, *p*-GSK3β, GSK3β, and GAPDH after transfection LY294002 inhibitor. (E) The ability of cell proliferation after transfection LY294002 inhibitor. Densitometric analyses of the band intensity ratios for GPD1/GAPDH, PI3K/GAPDH, PTEN/GAPDH, *p*-AKT/AKT, and *p*-GSK3β/GSK3β. Data are shown as the mean ± SD (n = 3, *p < 0.05, **p < 0.01, ***p < 0.001).

In addition, the expression levels of PI3K, AKT, *p*-AKT, and *p*-GSK3β were attenuated after treatment with LY294002, a PI3K/AKT pathway inhibitor (Fig. 4C, D and S11). The ability of cell proliferation was also partly recovered in breast cancer cells compared with the GPD1 overexpression group using the CCK-8 assay kit (Fig. 4E). These results indicated that GPD1 might increase lipid metabolism by activating PI3K/AKT signaling pathway.

### 3.5 GPD1 expression level in different populations of breast cancers

To further assess the potential clinical significance of GPD1, the Kaplan-Meier survival analysis was conducted in different populations of breast cancer using the UALCAN. The overall survival of GPD1 high expression level in breast cancer patients partially exceeded the populations of GPD1 low or medium expression level, although the results were not significant (Fig. 5A). Meanwhile, the survival of breast cancer was a significant difference between the GPD1 expression level and menopause status (*p* < 0.01, Fig. 5B). Moreover, the expression level of GPD1 existed an obvious change in different races of breast cancer patients (Fig. 5C, S5, S6). The median expression level of GPD1 in Asians at 0.607 was markedly lower than that of Caucasians at 1.842 and African-Americans at 1.249 (Table S6).

**Fig. 5:**
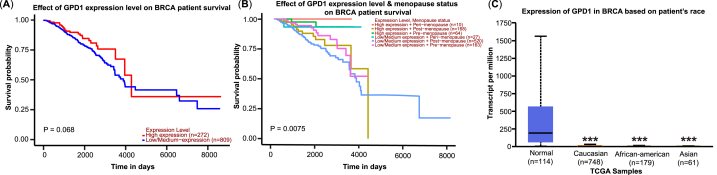
The clinical significance of GPD1 in different populations of breast cancers. (A) The overall survival at different expression levels of GPD1. (**B**) The Kaplan-Meier survival analysis between the GPD1 expression level and menopause status, including pre-menopause, peri-menopause, and post-menopause. (**C**) GPD1 expression levels in different breast cancer races.

## 4 Discussion

Breast cancer is considered to be a metabolic disease characterized by the accumulation of lipids and glycogen [22,23]. Relevant studies reported that GPD1 presented lower expression levels in the mRNA plane of breast cancer cells not only, but also in the protein plane of breast cancer tissues [18,19]. Liu et al. also demonstrated that GPD1 could inhibit the progression of renal clear cell carcinoma via lipid metabolism [11]. However, the GPD1-induced signaling pathway in breast cancer carcinogenesis was unknown. In this study, we found that GPD1 overexpression inhibited proliferation, migration, and invasion in breast cancer cells by activating PI3K/AKT signaling pathway and lipogenesis.

The elevated *p*-GSK3β expression level of the GPD1 overexpression group indicated that GPD1 inhibited glycogen synthesis in breast cancer cells (Fig. 3A). Meanwhile, the results of up-regulated lipid metabolism further elucidated that GPD1 overexpression promoted more glucose or metabolic intermediates converting into G3P, which is used to synthesize a variety of lipid metabolites [12,24]. However, the specific downstream regulatory mechanisms and in vivo experiments need to be further verified. Thus far, there have been many studies on lipid metabolism pathways and PI3K/AKT signaling pathways in breast cancer [25,26]. The PI3K/AKT signaling pathway could regulate lipid synthesis and metabolism via various target genes [8,26]. One study explained that hyperactive mutation of PI3K/AKT signaling protected breast cancer cells from oxidative stress and ferroptosis death through SREBP1/SCD1-mediated lipogenesis [27]. Zhang et al. clarified that FASN and MGLL regulated cancer cell invasion and metastasis, immune microenvironment, and epithelial-mesenchymal transition via PI3K/AKT-mediated free fatty acids metabolism pathway [28]. Although the PI3K/AKT signaling pathway had different effects on promoting or inhibiting tumor cells, we mainly elucidate the intracellular changes in breast cancer from the perspective of substances metabolism, which may be related to the accumulation of intermediate secondary products or autophagy (data unpublished). Cellular lipids, including phospholipids, fatty acids, and cholesterol, play a key role as essential components of membranes, signaling molecules, nutrient storage, and unique disease biomarkers [7,9].

Among these lipids, cholesterol is the basic substance for the synthesis of estrogen [9]. As shown in Fig. 3B, S3, S4, and Table S4, steroid biosynthesis and cholesterol metabolism had a significant change at different degrees. And the estrogen levels were strongly correlated with survival in breast cancer patients by the TCGA database (Fig. 5B) [[29], [30], [31]]. The survival probability of pre-menopause was higher than post-menopause in GPD1 low/medium expression levels not only, but also in GPD1 high expression levels. The survival probability of GPD1 high expression level also exceeded the GPD1 low/medium expression level in pre-menopause. However, the clinical relationship between estrogen levels and GPD1 expression levels needs further study. Furthermore, the survival probability of the GPD1 high expression level was remarkedly advanced compared with the GPD1 low/medium expression level in the Luminal subtype (Fig. S7) [32,33]. Meanwhile, the protein expression level of GPD1 in the Luminal subtype was highest compared to the HER2+ subtype and TNBC subtype [19].

The above study indicated that the relationship between GPD1 expression level and survival rate presents a positive correlation in breast cancer patients. The GPD1 maybe act as a tumor-suppressor gene and be used for the early diagnostic or prognostic biomarkers [18,19,34]. In addition, some studies demonstrated that phosphatidylcholines are also regarded as a prognostic biomarker of breast cancer development according to 1624 first primary incident invasive breast cancer cases [35,36]. Similarly, elevated phosphatidylethanolamine has been observed in breast cancer almost as consistently as increased phosphatidylcholines [37]. Compared to single biomarkers or traditional tumor staging, the composite multi-molecule models are superior in identifying high-risk patients or predicting prognostic survival [2,3,38]. To improve the prognostic accuracy of GPD1, the composite biomarker models included several lipid metabolites correlated with GPD1, such as estrogen, free fatty acids, cholesterol, triglyceride, and phospholipids.

## 5 Conclusions

In summary, our study confirmed that GPD1 inhibits the ability of proliferation, migration, and invasion in breast cancer cells via activating the PI3K/AKT-mediated lipid signaling pathway. Meanwhile, the expression level of GPD1 has a positive correlation with lipid metabolites and the survival rate of breast cancer patients, respectively. This study reveals a new mechanism underlying the tumor suppression ability of GPD1 and provides a new perspective on forming composite prognostic biomarkers with GPD1 and lipid metabolites. The results of this study have the potential to be used in the development of novel therapies for breast cancer.

## Author Contributions Statement

**Zhengchao Xia**: Methodology, Project administration, Writing- original draft. **Ningming Zhao**: Data curation. **Mingzhou Liu**: Formal analysis. **Dandan Jiang**: Data curation, Investigation. **Shanjun Gao**: Software, Validation. **Peizhi Ma**: Conceptualization. **Li Huang**: Funding acquisition, Writing-review and editing.

## Data availability statement

Data included in article/supp. material/referenced in article.

## Declaration of competing interest

The authors declare that they have no known competing financial interests or personal relationships that could have appeared to influence the work reported in this paper.

## Figures and Tables

**figs1:**
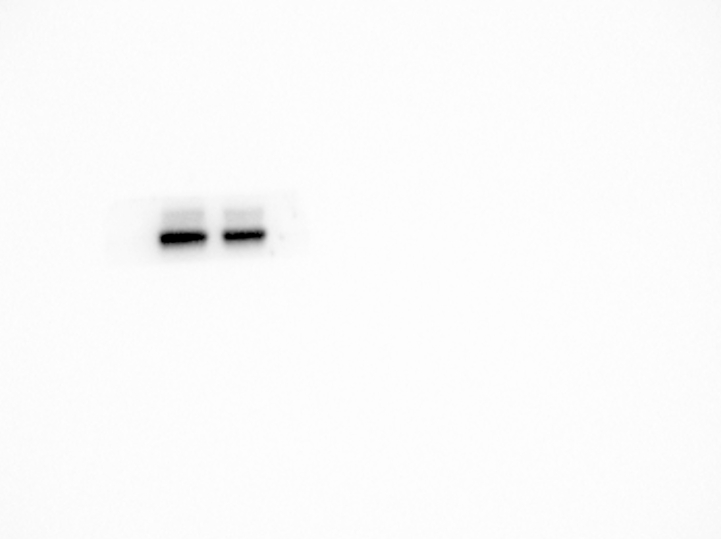


**figs2:**
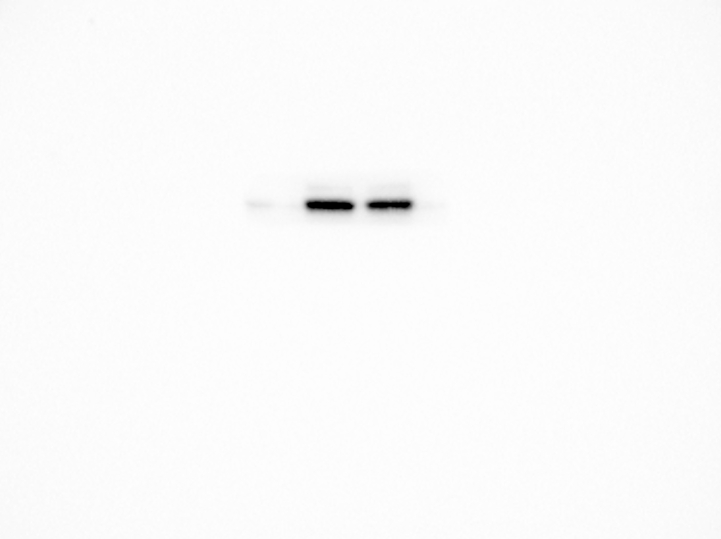


**figs3:**
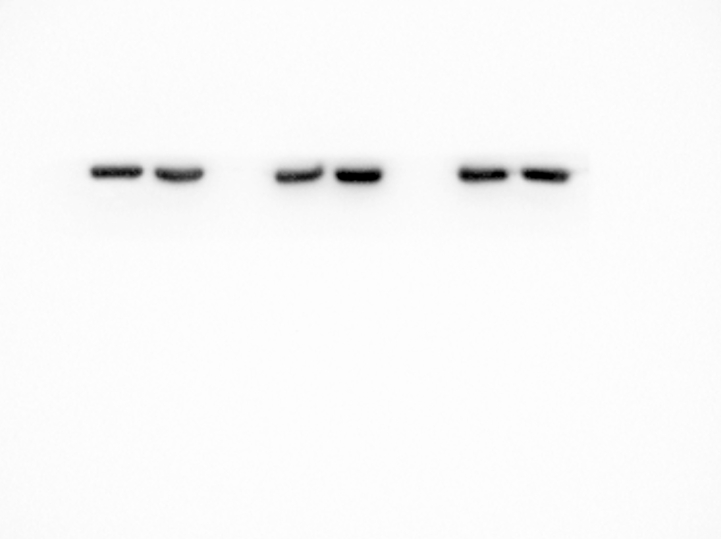


**figs4:**
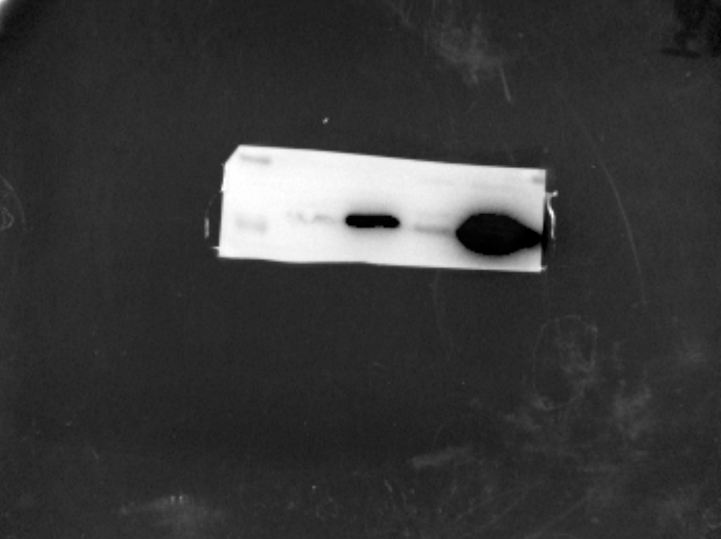


**figs5:**
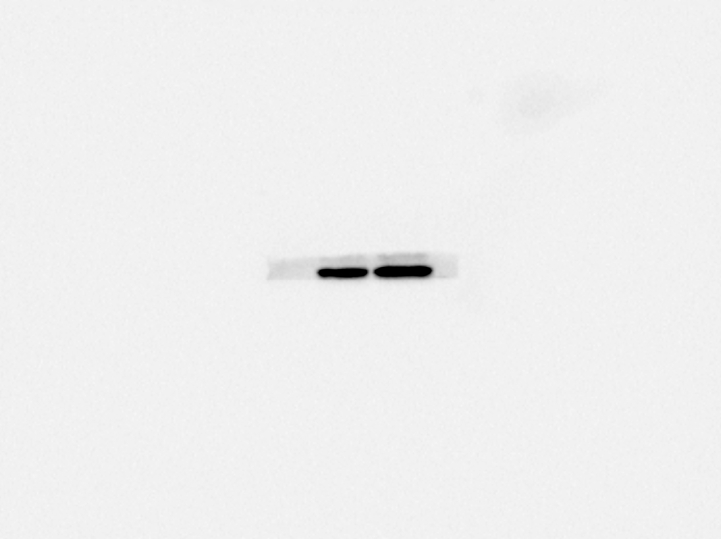


**figs6:**
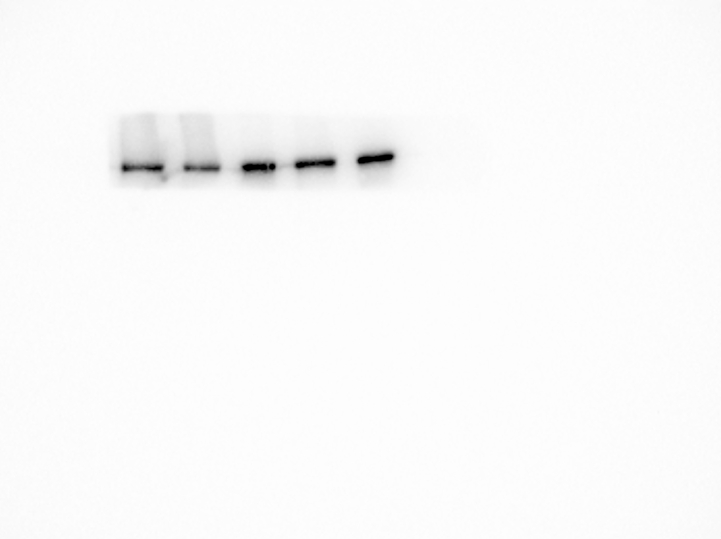


**figs7:**
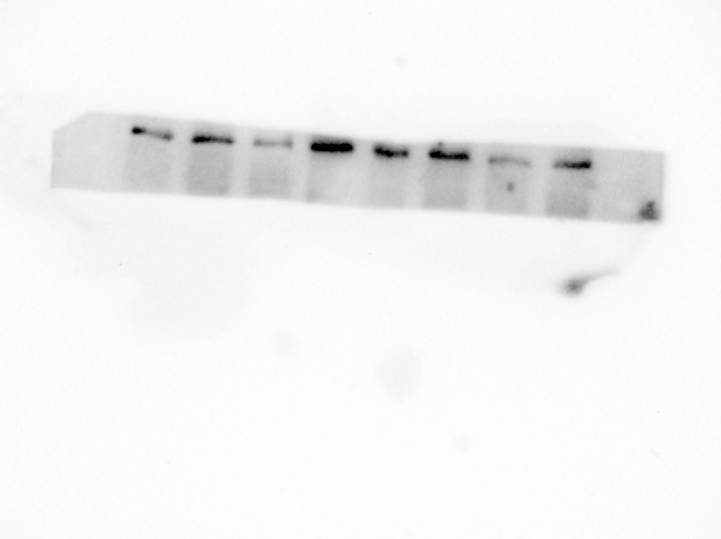


**figs8:**
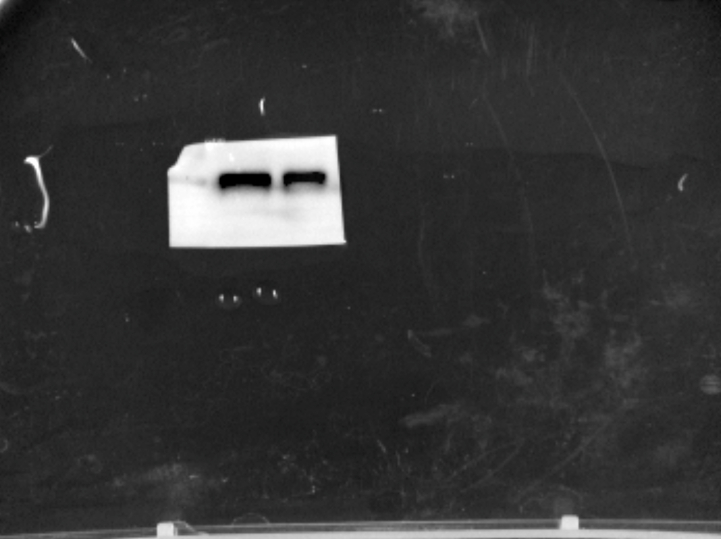


**figs9:**
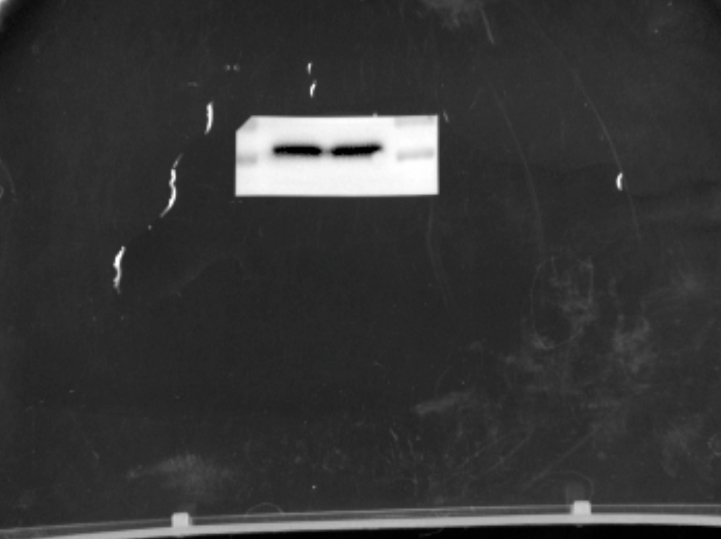


**figs10:**
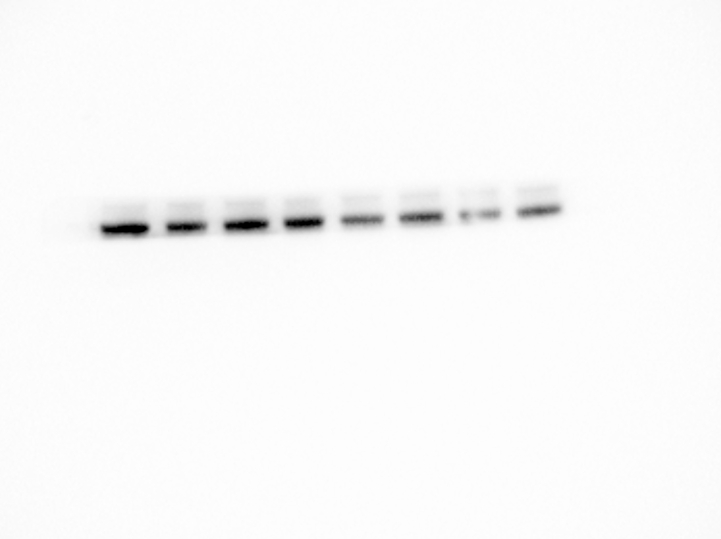


**figs11:**
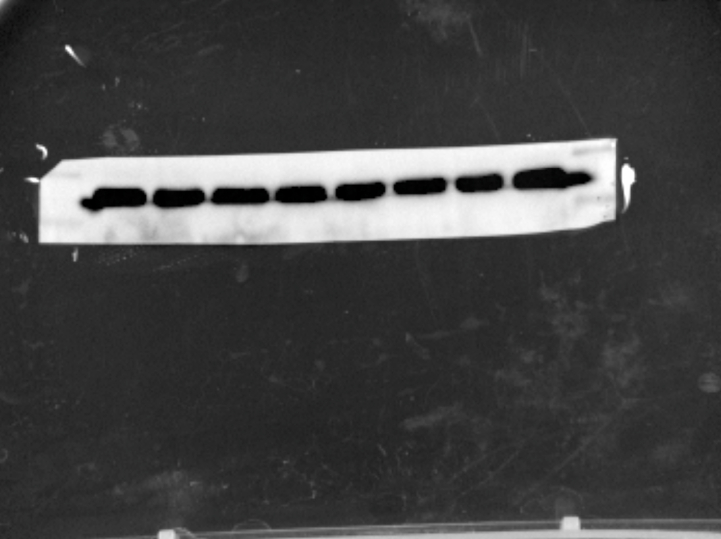


**figs12:**
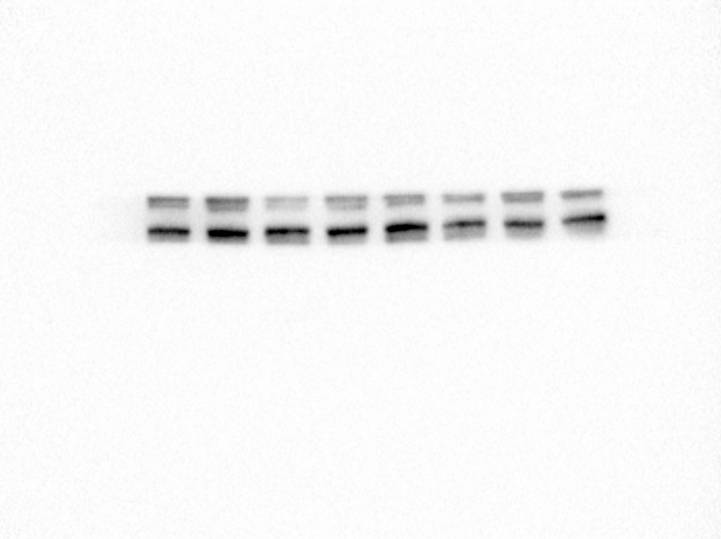


**figs13:**
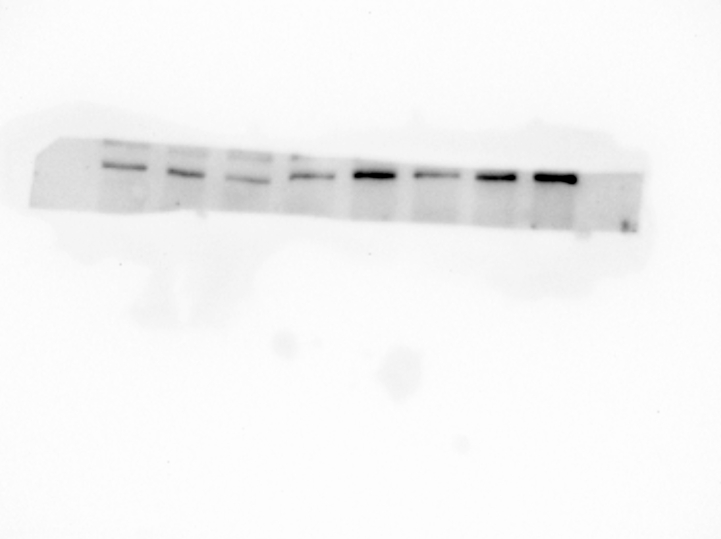


**figs14:**
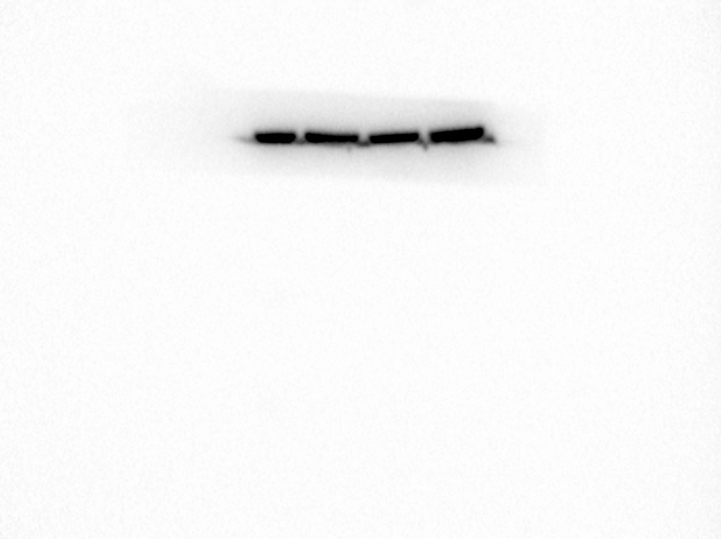


**figs15:**
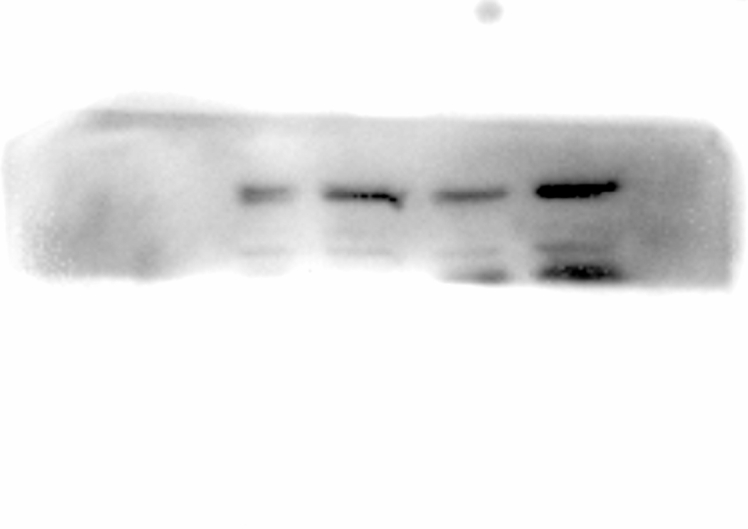


## References

[1] Sung H. (2021). Global cancer statistics 2020: GLOBOCAN estimates of incidence and mortality worldwide for 36 cancers in 185 countries. CA. Cancer J. Clin..

[2] Barzaman K. (2020). Breast cancer: biology, biomarkers, and treatments. Int. Immunopharm..

[3] Jafari S.H. (2018). Breast cancer diagnosis: imaging techniques and biochemical markers. J. Cell. Physiol..

[4] Mueller C., Haymond A., Davis J.B., Williams A., Espina V. (2018). Protein biomarkers for subtyping breast cancer and implications for future research. Expert Rev. Proteomics.

[5] Harbeck N., Gnant M. (2017). Breast cancer. Lancet.

[6] Wu Z., Wu J., Zhao Q., Fu S., Jin J. (2020). Emerging roles of aerobic glycolysis in breast cancer. Clin. Transl. Oncol..

[7] Ward A.V., Anderson S.M., Sartorius C.A. (2021). Advances in analyzing the breast cancer lipidome and its relevance to disease progression and treatment. J. Mammary Gland Biol. Neoplasia.

[8] Miricescu D. (2021). PI3K/AKT/mTOR signaling pathway in breast cancer: from molecular landscape to clinical aspects. Int. J. Mol. Sci..

[9] Butler L. (2020). Lipids and cancer: emerging roles in pathogenesis, diagnosis and therapeutic intervention. Adv. Drug Deliv. Rev..

[10] Xia Z. (2019). Multiple-omics techniques reveal the role of glycerophospholipid metabolic pathway in the response of saccharomyces cerevisiaeagainst hypoxic stress. Front. Microbiol..

[11] Liu R. (2021). A HIF1α-GPD1 feedforward loop inhibits the progression of renal clear cell carcinoma via mitochondrial function and lipid metabolism. J. Exp. Clin. Cancer Res..

[12] Ocaña M.C., Martínez-Poveda B., Quesada A.R., Medina M.Á. (2020). Glucose favors lipid anabolic metabolism in the invasive breast cancer cell line MDA-MB-231. Biology.

[13] Lin H. (2021). Case report: identification of a novel homozygous mutation in GPD1 gene of a Chinese child with transient infantile hypertriglyceridemia. Front. Genet..

[14] Li N. (2017). Biallelic mutations in GPD1 gene in a Chinese boy mainly presented with obesity, insulin resistance, fatty liver, and short stature. Am. J. Med. Genet..

[15] Xie J. (2020). GPD1 enhances the anticancer effects of metformin by synergistically increasing total cellular glycerol-3-phosphate. Cancer Res..

[16] Rusu P. (2019). GPD1 specifically marks dormant glioma stem cells with a distinct metabolic profile. Cell Stem Cell.

[17] Zhang W. (2022). Allosteric activation of the metabolic enzyme GPD1 inhibits bladder cancer growth via the lysoPC-PAFR-TRPV2 axis. J. Hematol. Oncol..

[18] Zhou C. (2017). Identification of glycerol-3-phosphate dehydrogenase 1 as a tumour suppressor in human breast cancer. Oncotarget.

[19] Yoneten K.K. (2019). Comparative proteome analysis of breast cancer tissues highlights the importance of glycerol-3phosphate dehydrogenase 1 and monoacylglycerol lipase in breast cancer metabolism. Cancer Genomics Proteomics.

[20] Liao X. (2018). LAMP3 regulates hepatic lipid metabolism through activating PI3K/Akt pathway. Mol. Cell. Endocrinol..

[21] Chen J. (2019). HIF-2α upregulation mediated by hypoxia promotes NAFLD-HCC progression by activating lipid synthesis via the PI3K-AKT-mTOR pathway. Aging (Albany NY).

[22] Cantor J.R., Sabatini D.M. (2012). Cancer cell metabolism: one hallmark, many faces. Cancer Discov..

[23] Ganapathy-Kanniappan S. (2018). Molecular intricacies of aerobic glycolysis in cancer: current insights into the classic metabolic phenotype. Crit. Rev. Biochem. Mol. Biol..

[24] Sato T., Morita A., Mori N., Miura S. (2014). The role of glycerol-3-phosphate dehydrogenase 1 in the progression of fatty liver after acute ethanol administration in mice. Biochem. Biophys. Res. Commun..

[25] Eiriksson F.F. (2020). Lipidomic study of cell lines reveals differences between breast cancer subtypes. PLoS One.

[26] Alzahrani A.S. (2019). PI3K/Akt/mTOR inhibitors in cancer: at the bench and bedside. Semin. Cancer Biol..

[27] Yi J., Zhu J., Wu J., Thompson C.B., Jiang X. (2020). Oncogenic activation of PI3K-AKT-mTOR signaling suppresses ferroptosis via SREBP-mediated lipogenesis. Proc. Natl. Acad. Sci. U.S.A..

[28] Zhang J., Song Y., Shi Q., Fu L. (2021). Research progress on FASN and MGLL in the regulation of abnormal lipid metabolism and the relationship between tumor invasion and metastasis. Front. Med..

[29] Chlebowski R.T. (2020). Association of menopausal hormone therapy with breast cancer incidence and mortality during long-term follow-up of the women's health initiative randomized clinical trials. JAMA.

[30] Ward A.V. (2022). Estrogens and progestins cooperatively shift breast cancer cell metabolism. Cancers.

[31] Deng Y., Huang H., Shi J., Jin H. (2022). Identification of candidate genes in breast cancer induced by estrogen plus progestogens using bioinformatic analysis. Int. J. Mol. Sci..

[32] Howlader N., Cronin K.A., Kurian A.W., Andridge R. (2018). Differences in breast cancer survival by molecular subtypes in the United States. Cancer Epidemiol. Biomarkers Prev..

[33] Waks A.G., Winer E.P. (2019). Breast cancer treatment: a review. JAMA.

[34] Xia R. (2021). Prognostic value of a novel glycolysis-related gene expression signature for gastrointestinal cancer in the Asian population. Cancer Cell Int..

[35] His M. (2019). Prospective analysis of circulating metabolites and breast cancer in EPIC. BMC Med..

[36] Guo R. (2020). The function and mechanism of lipid molecules and their roles in the diagnosis and prognosis of breast cancer. Molecules.

[37] Shah T. (2018). Molecular causes of elevated phosphoethanolamine in breast and pancreatic cancer cells. NMR Biomed..

[38] Zou Y. (2021). Breath profile as composite biomarkers for lung cancer diagnosis. Lung Cancer.

