# Supplementary material for: GPD1 inhibits the carcinogenesis of breast cancer through increasing PI3K/AKT-mediated lipid metabolism signaling pathway
Source: Heliyon. 2023 Jul 8;9(7):e18128. doi: 10.1016/j.heliyon.2023.e18128 (PMC10362286; doi:10.1016/j.heliyon.2023.e18128)
Supplement: Multimedia component 1 [file mmc1.docx]

***Supplementary Material***

**GPD1 inhibits the carcinogenesis of breast cancer through increasing PI3K/AKT-mediated lipid metabolism signaling pathway**

Zhengchao Xia^1^, Ningming Zhao^1^, Mingzhou Liu^1^, DanDan Jiang^1^, Shanjun Gao^2^, Peizhi Ma^1*^, Li Huang^3*^

*** Correspondence:**

Peizhi Ma, mpeizhi@163.com

Li Huang, huangli2019@zzu.edu.cn

**The full details of the lipidomics methods**

The analytical conditions were as follows, UPLC: column, Thermo Accucore™ C30 (2.6 μm, 2.1 mm*100 mm); solvent system, A: acetonitrile/water (60/40, V/V, 0.1% formic acid, 10 mmol/L ammonium formate), B: acetonitrile/isopropanol (10/90, 0.1% formic acid, 10 mmol/L ammonium formate); gradient program, A/B (80:20) at 0 min, 70:30 at 2.0 min, 40:60 at 4 min, 15:85 at 9 min, 10:90 at 14 min, 5:95 at 15.5 min, 5:95 at 17.3 min, 80:20 at 17.3 min, 80:20 at 20 min; flow rate, 0.35 ml/min; temperature, 45 °C; injection volume: 2μl. The effluent was alternatively connected to an ESI-triple quadrupole-linear ion trap (QTRAP)-MS.

LIT and triple quadrupole (QQQ) scans were acquired on a triple quadrupole-linear ion trap mass spectrometer (QTRAP), QTRAP® LC-MS/MS System, equipped with an ESI Turbo Ion-Spray interface, operating in positive and negative ion mode and controlled by Analyst 1.6.3 software (Sciex). The ESI source operation parameters were as follows: ion source, turbo spray; source temperature 500 °C; ion spray voltage (IS) 5500 V (Positive), -4500 V (Negative); ion source gas 1 (GS1), gas 2 (GS2), curtain gas (CUR) were set at 45, 55, and 35 psi, respectively; the collision gas (CAD) was medium. Instrument tuning and mass calibration were performed with 10 and 100 μmol/L polypropylene glycol solutions in QQQ and LIT modes, respectively. QQQ scans were acquired as MRM experiments with collision gas (nitrogen) set to 5 psi. DP and CE for individual MRM transitions were done with further DP and CE optimization. A specific set of MRM transitions were monitored for each period according to the metabolites eluted within this period.

Principal component analysis (PCA) and orthogonal partial least-squares discriminant analysis (OPLS-DA) were performed using the R package. The hierarchical cluster analysis (HCA) and Pearson correlation coefficients (PCC) results of metabolites were presented as heatmaps with dendrograms. VIP values were extracted from the OPLS-DA result, which also contains score plots and permutation plots, and were generated using MetaboAnalystR. The data was log transform (log2) and mean centering before OPLS-DA.

**Quantitative analysis of cell migration assay**

First, the image format is converted to 8-bit by Type in the Image. Then, the image increased contrast by Enhance Contrast (Saturated pixels: 6%, normalize), smoothing, and highlighting the cell edges by Find Edges in Process. Finally, the area of migration was displayed by regulating the threshold by Adjust in Image, and measured the area in Analyze after using the magic wand tool.

**Quantitative analysis of western blotting**

Firstly, the image format is converted to 8-bit by Type in the Image. Secondly, the peak area of bands was shown by Select First Lane and Plot Lanes in Gels of Analyze. Thirdly, we split the individual peaks with a line tool and measure the area with the magic wand tool.

**Supplementary Tables**

**Table S1**. The sequences of all the primers.

| **Name** | **Oligonucleotide sequence (5' - 3')** |
| --- | --- |
| GPD1 | F: GCCATCTGAAGGCAAACGC |
|  | R: GCCAATGGTTGTCTCACAGAAC |
| LPIAT | F: GCCCTCCCTGATGGAGACA |
|  | R: GTAGGTGCGGTAGCGGAAGA |
| LPCAT3 | F: CTAGCTAGCCACCATGGATT |
|  | R: CCGCTCGAGCTAGTCCGCTTTCT |
| CEPT1 | F: CCTCCTTTTCTGGTCCTGTTTGAT |
|  | R: TATCTTTAGTTAAAATGACCCCAC |
| FASN | F: GGGTTTAGAGATGGGTGTTAAT |
|  | R: CCCACACCTAACAACCTTAAAC |
| ACC1 | F: TACAAACGCAAGAGTCATACTGG |
|  | R: CTTTCCAATTCAAGGTTCTGAC |
| SREBP1 | F: ACAGTGACTTCCCTGGCCTAT |
|  | R: GCATGGACGGGTACATCTTCAA |
| GAPDH | F: AGCCACATCGCTCAGACAC |
|  | R: GCCCAATACGACCAAATCC |

**Table S2**. The antibodies of all proteins.

| **Name** | **Description** | **Product code** | **Lot number** |
| --- | --- | --- | --- |
| GPD1 | rabbit polyclonal | 13451 | 00063207 |
| PI3 Kinase P110β | mouse monoclonal | 67121 | 10016961 |
| AKT | rabbit polyclonal | 10176 | 00106540 |
| phospho-AKT (Ser473) | mouse monoclonal | 66444 | 10022023 |
| GSK3β | rabbit polyclonal | 22104 | 00096178 |
| phospho-GSK3β (Ser9) | mouse monoclonal | 67558 | 10016184 |
| PTEN | rabbit polyclonal | 22034 | 00101141 |
| GAPDH | mouse monoclonal | 60004 | 10020246 |

**Table S3**. The annotated information of all metabolites in HMDB.

The table contains 1310 lipid metabolites. It includes formula of each compounds, primary (**GP**: glycerophospholipids, **GL**: glycerides, **SP**: sphingolipids, **FA**: fatty acyls, **ST**: sterol lipids, **PR**: prenol lipids) and secondary classification, the peak area of mass spectrum, the ID number of CPD and HMDB database.

**Table S4**. The related information of 110 differential metabolites.

Table S4 contains 110 differential lipid metabolites with VIP and Fold change. It also includes formula, primary (GP, GL, SP, FA, ST, PR) and secondary classification, the peak area of mass spectrum, the VIP, p-value, fold change, log2FC and up-/down-regulated type.

**Table S5**. The KEGG enrichment analysis of different metabolites.

This table shows the KEGG pathway, the ID number of ko, the number of differential metabolites enrichment and all compound in each pathway, the index of detailed differential metabolites.

**Table S6**. The expression level of GPD1 in breast cancer based on patient’s race.

| **TCGA samples** | **Series** | | | | |
| --- | --- | --- | --- | --- | --- |
|  | **Low** | **Q1** | **Median** | **Q3** | **High** |
| **Normal (n=114)** | 1.386 | 66.627 | 192.534 | 557.5 | 1561.279 |
| **Caucasian (n=748)** | 0 | 0.475 | 1.842 | 7.599 | 32.326 |
| **African-American (n=179)** | 0.022 | 0.361 | 1.249 | 3.116 | 12.748 |
| **Asian (n=61)** | 0.021 | 0.201 | 0.607 | 1.591 | 8.521 |

Normal: the people without breast cancer.

**Supplementary Figures**


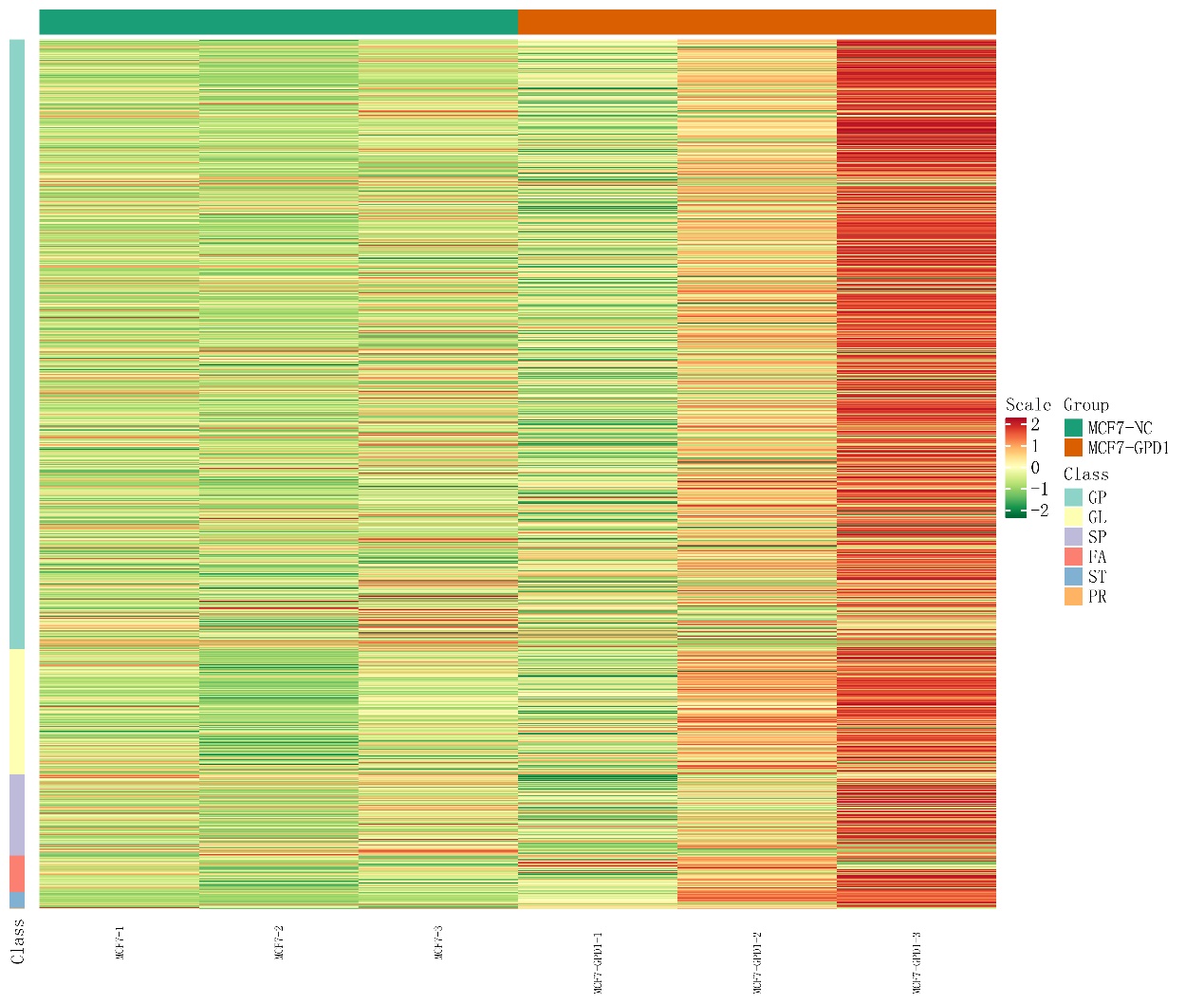


**Figure S1**. The cluster analysis heatmap of 1310 lipid metabolites.

The figure showed 1310 lipid metabolites, included glycerophospholipids (**GP**), glycerides (**GL**), sphingolipids (**SP**), fatty acyls (**FA**), sterol lipids (**ST**), prenol lipids (**PR**). Red and green indicated high and low expression, respectively.


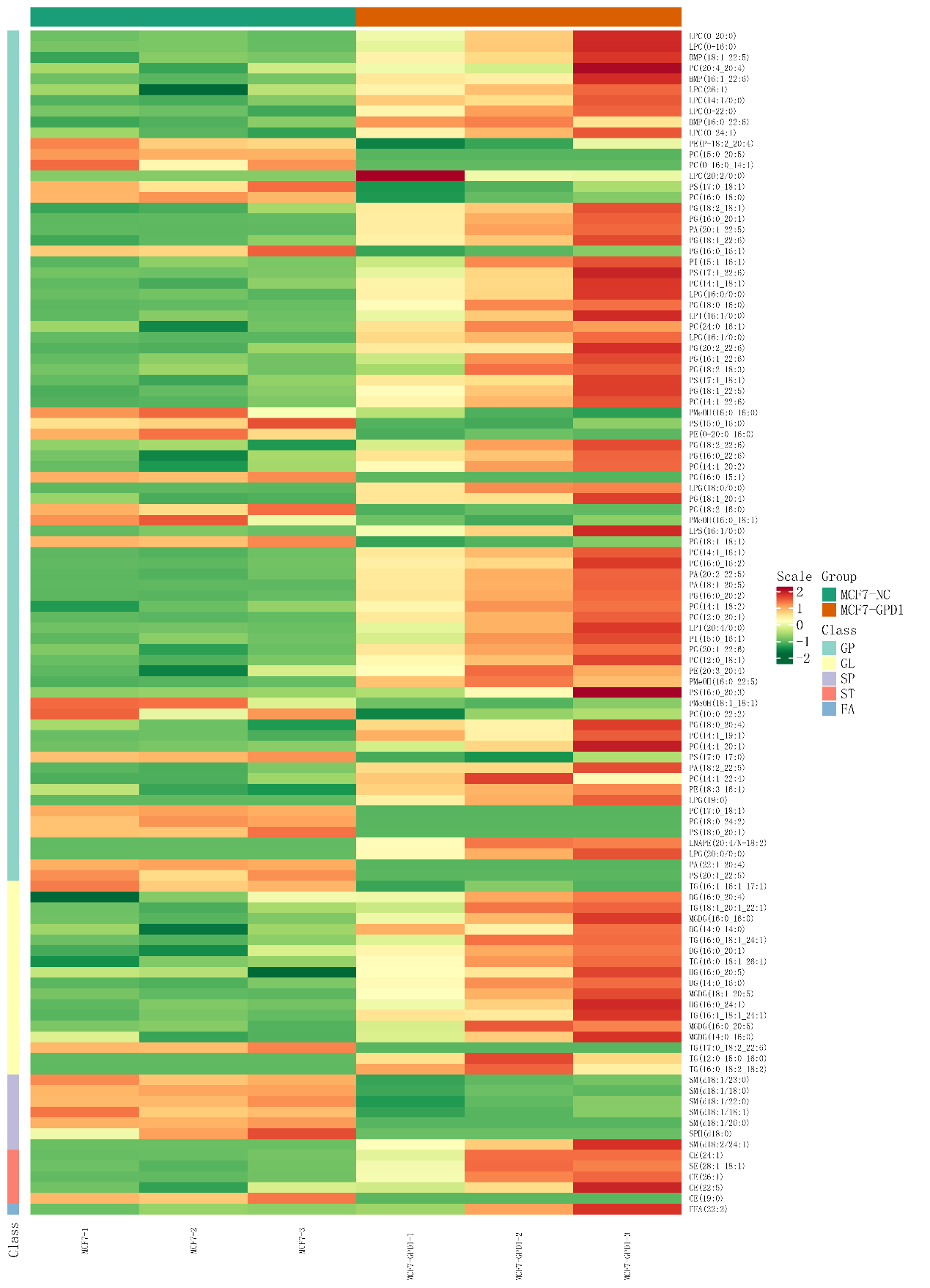


**Figure S2.** The cluster analysis heatmap of differential metabolites.

This figure showed 110 differential metabolites (DMs), included glycerophospholipids (**GP**), glycerides (**GL**), sphingolipids (**SP**), fatty acyls (**FA**), sterol lipids (**ST**), prenol lipids (**PR**). The right side presented the primary classification of lipid metabolites, included the number of carbon atoms and lipid unsaturation. Red and green indicated high and low expression, respectively.


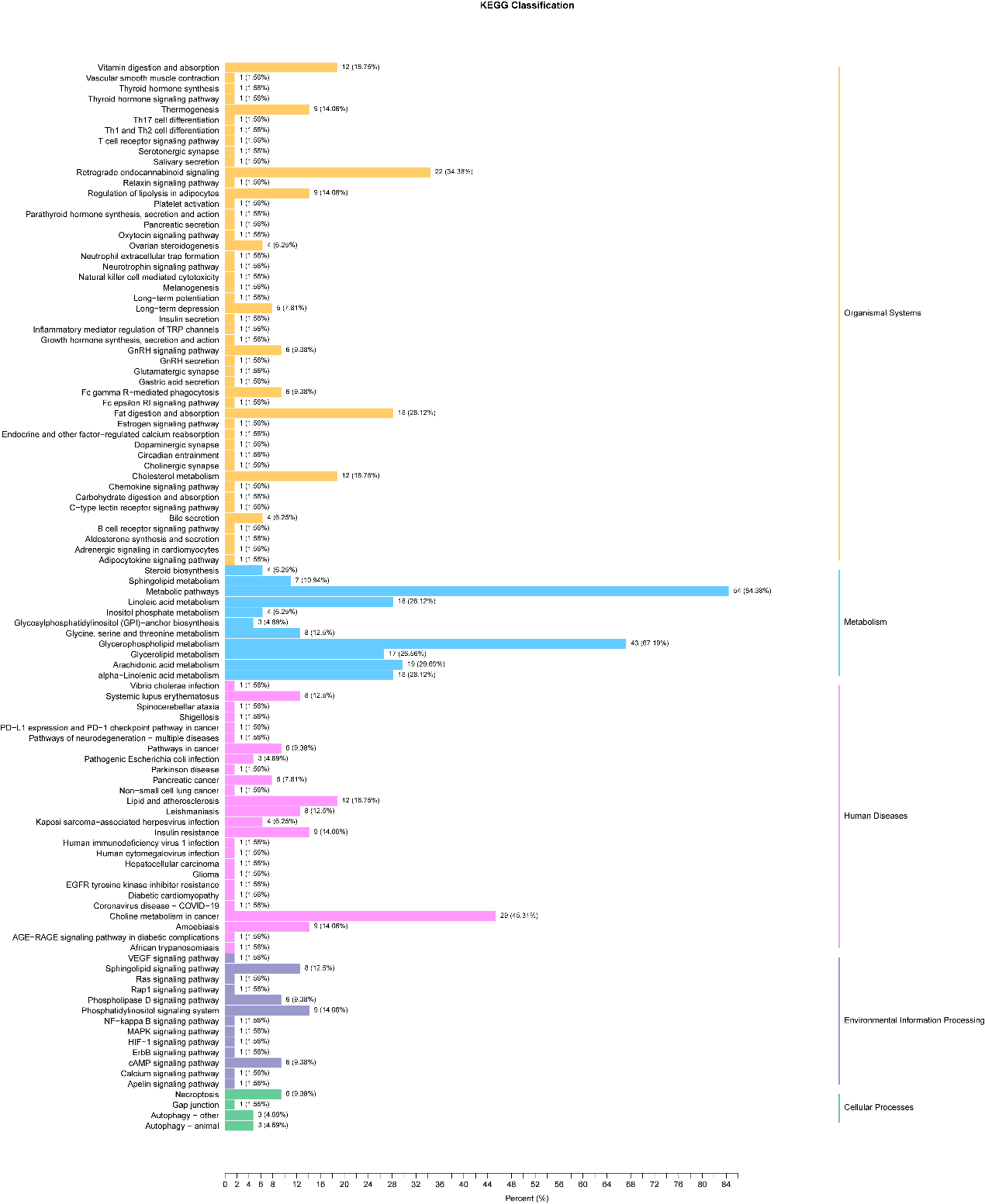


**Figure S3**. The KEGG classification plot of differential metabolites.

The figure displayed the KEGG annotations and pathway enrichment analysis of differential metabolites. The left side showed the name of each pathway. The numbers in the histogram indicated the number and proportion of differential metabolites enriched into this pathway.


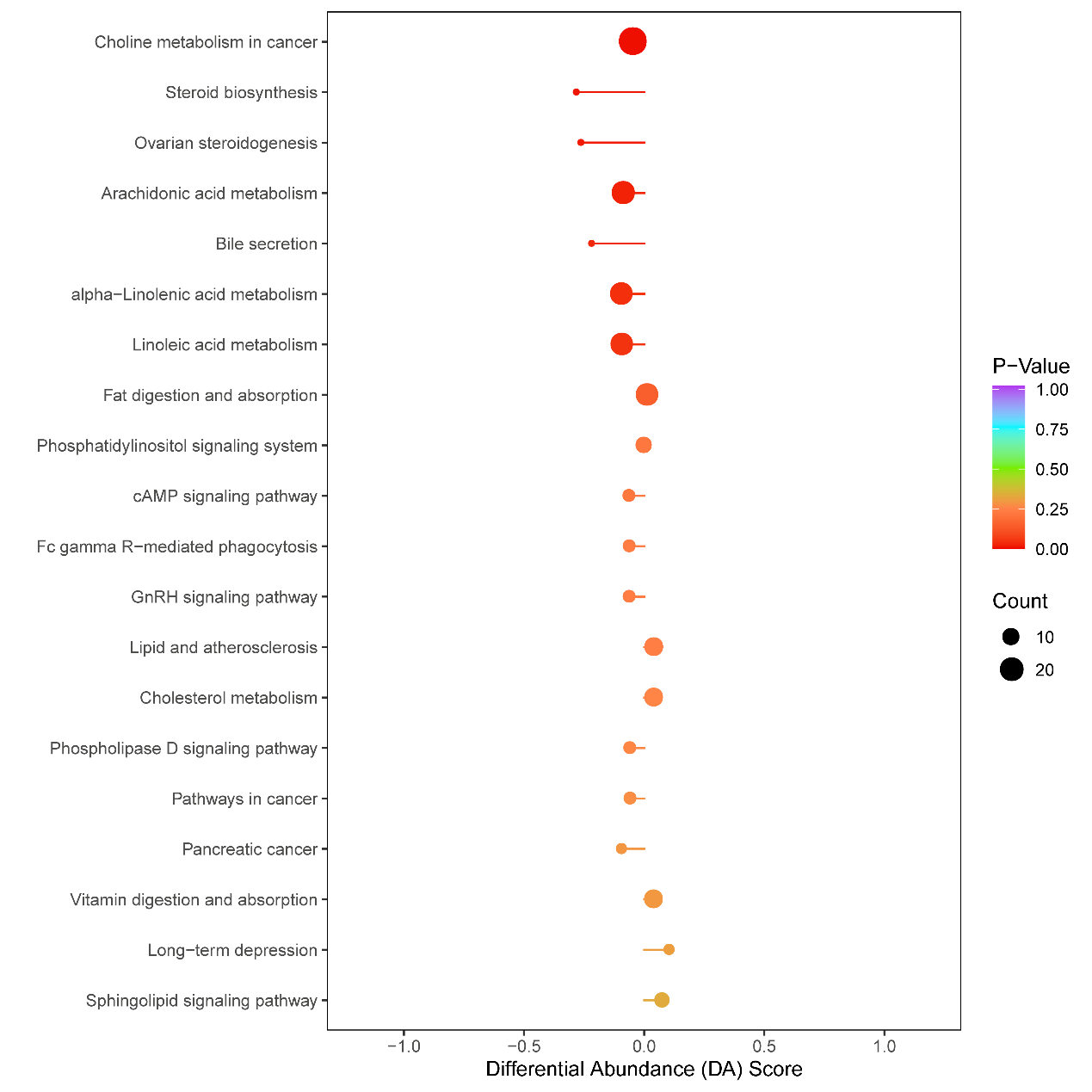


**Figure S4**. The differential abundance (DA) score plot of differential metabolites.

The ordinate represented the name of difference pathway, and the abscissa represented the DA score. The DA score reflected the overall change in all metabolites of the metabolic pathway. Score 1 and -1 indicated up- or down-regulated trends in differential metabolite expression in this pathway, respectively. The segment length indicated the absolute value of DA score. The dot size and color indicated the number of differential metabolites and in this pathway the P-value.


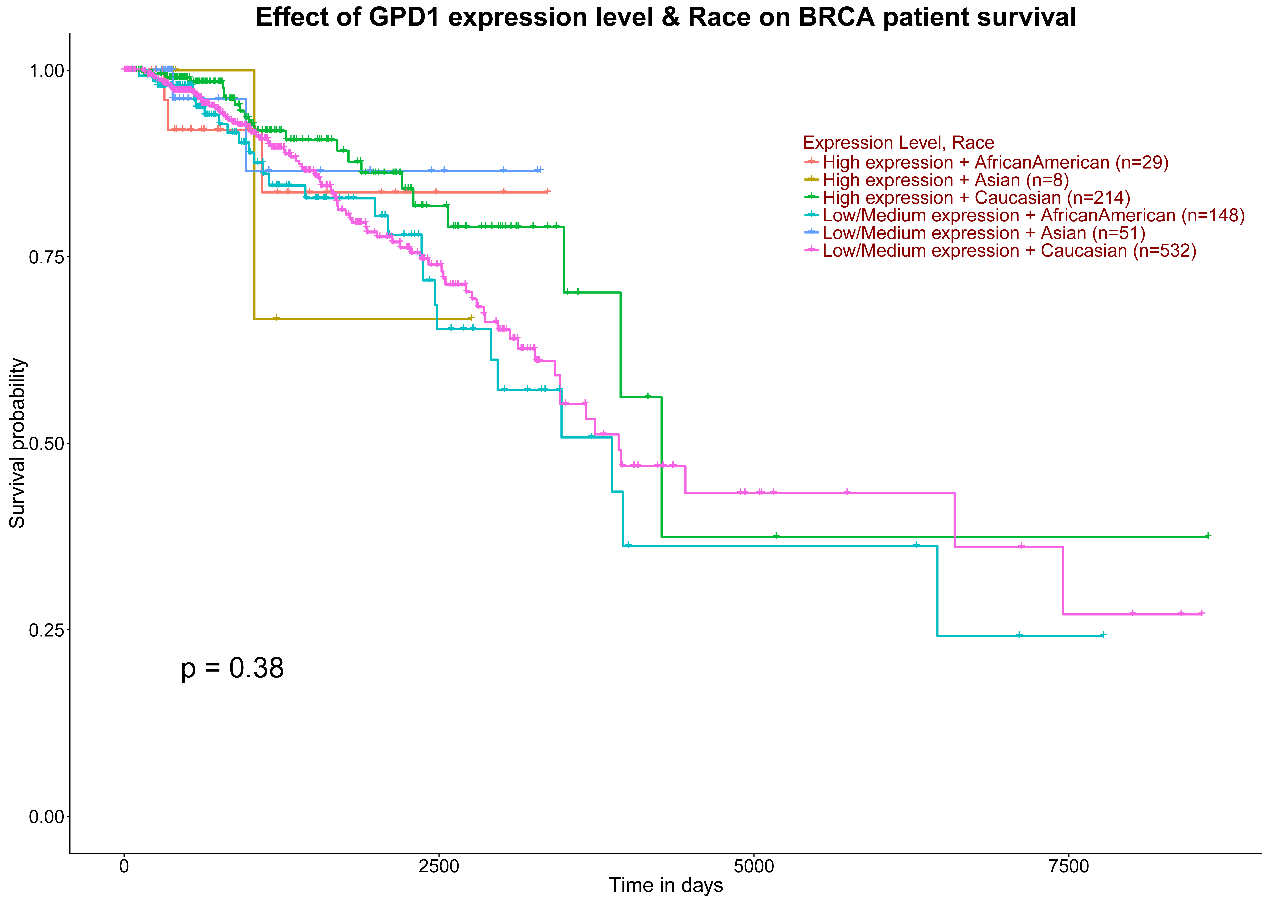


**Figure S5.** The survival relationship between GPD1 expression level and race in breast cancer patients.


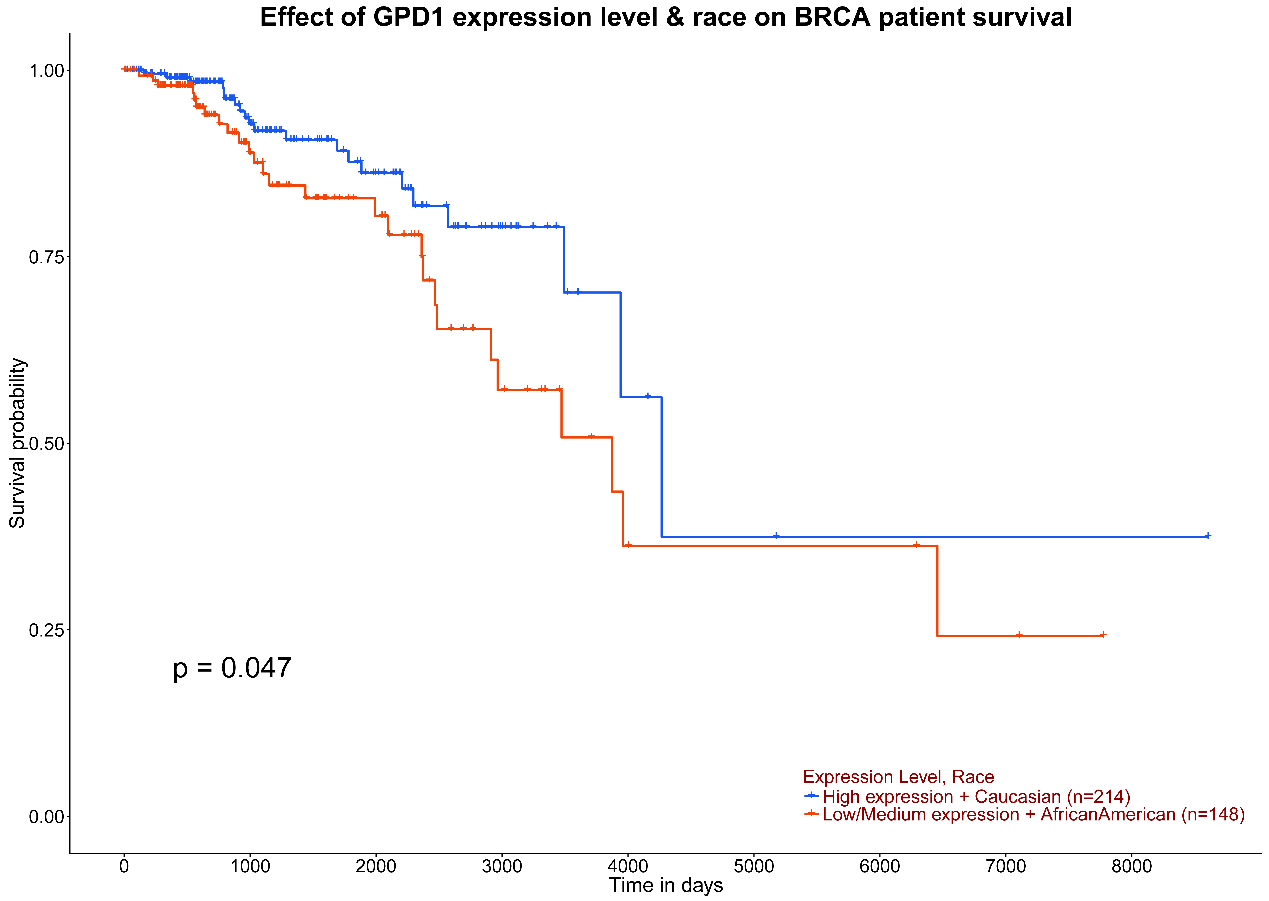


**Figure S6**. The survival probability between Caucasian of GPD1 high expression and African-American of GPD1 low/medium expression in breast cancer patients.


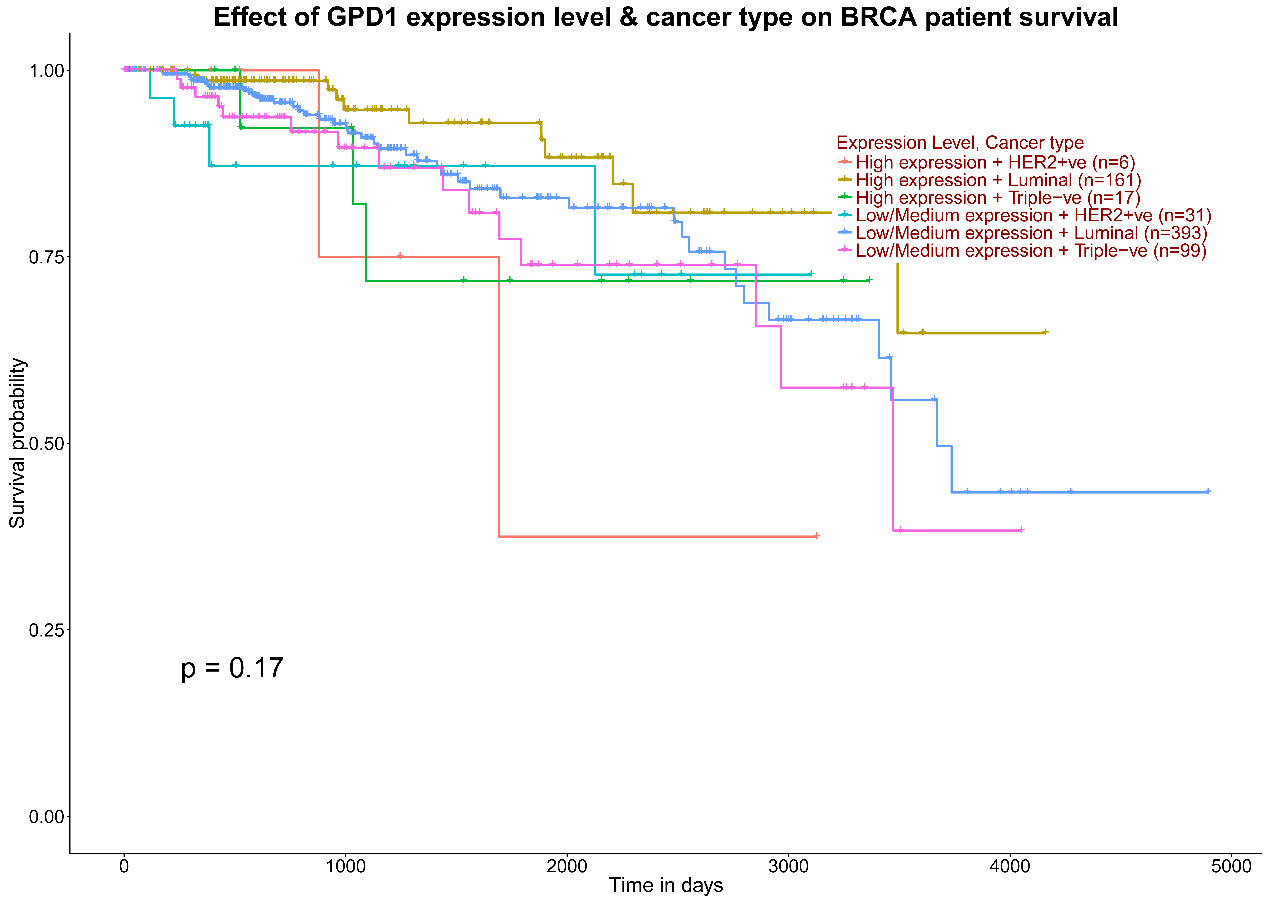


**Figure S7**. The survival probability between GPD1 expression level and cancer subtype in breast cancer patients.

In the Luminal subtype, the survival probability of the GPD1 high expression level (yellow line) was advanced the GPD1 low/medium expression level (blue line).


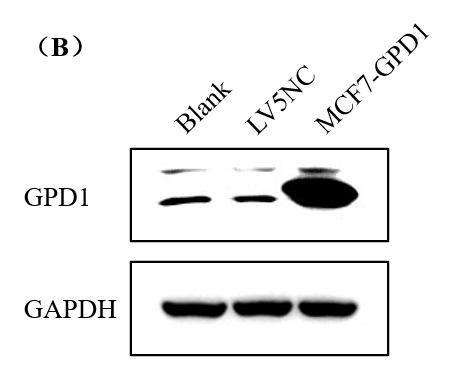


**Figure S8**. The mRNA level and protein level of GPD1 overexpression in MCF-7 cell lines by lentiviral stable transfection. The expression level of GAPDH had not changed in transcription and translation process. **(A)** The mRNA levels. **(B-C)** The protein level. Blank: MCF-7 cell lines; LV5NC: lentiviral negative control. MCF7-GPD1: GPD1 overexpression cell lines. Data are shown as the mean ± SD (n = 3, ****p* < 0.001).

**Figure S9**. The uncropped image of figure 2C.

The first strip of image was used to reconfirmed the low expression of MDA-MB-231 cell. The second to fourth strips were MCF-7, MDA-MB-231 and MCF-10A, respectively.

**Figure S10**. The original images of figure 4A.

The first to fourth strips of fig.S10-GPD1 were 231 cells and MCF-7 cells, respectively. The first to second strips of fig.S10-PI3K were MCF-7 cells, and the fifth to sixth strips of fig.S10-PI3K were 231 cells. The first to second strips of fig.S10-p-AKT were MCF7-NC cells, and the third to fifth strips were MCF7-GPD1 cells. The first to second strips of fig.S10-GSK3β were MCF-7 cells, and the third to eighth strips of fig.S10-GSK3β were GSK3β of figure 4C.

**Figure S11**. The original images of figure 4C.

The first to sixth strips of fig.S11-PI3K, p-AKT, AKT, p-GSK3β and GAPDH were MCF-7 cells and 231 cells, respectively. The third to eighth strips of fig.S11-GSK3β were MCF-7 cells and 231 cells, respectively.
